# Development of a Novel Loop-Mediated Isothermal Amplification Method for the Rapid Detection of Monkeypox Virus Infections

**DOI:** 10.3390/v15010084

**Published:** 2022-12-28

**Authors:** Chao Yu, Lulu Zuo, Jing Miao, Lingjing Mao, Benjamin Selekon, Ella Gonofio, Emmanuel Nakoune, Nicolas Berthet, Gary Wong

**Affiliations:** 1 Viral Hemorrhagic Fevers Research Unit, CAS Key Laboratory of Molecular Virology and Immunology, Institut Pasteur of Shanghai, Chinese Academy of Sciences, Shanghai 200031, China; 2 University of Chinese Academy of Sciences, Beijing 100049, China; 3 Centre for Microbes, Development, and Health, Institut Pasteur of Shanghai, Chinese Academy of Sciences, Unit of Discovery and Molecular Characterization of Pathogens, Shanghai 200031, China; 4 Laboratory of Arboviruses, Viral Hemorrhagic Fevers, Emerging viruses and Zoonoses, Institut Pasteur of Bangui, Bangui P.O. Box 923, Central African Republic; 5 Institut Pasteur, Unité Environnement et Risque Infectieux, Cellule d’Intervention Biologique d’Urgence, 75724 Paris, France

**Keywords:** monkeypox virus, molecular diagnosis, isothermal amplification, loop-mediated isothermal amplification, visual detection

## Abstract

A recent outbreak of monkeypox virus (mpox) has prompted researchers to explore diagnostics as a means of impeding transmission and further spread. Rapid, sensitive, and specific methods are crucial for accurately diagnosing mpox infections. Here, we developed a loop-mediated isothermal amplification (LAMP) assay for the specific detection of mpox. The primer sets were designed to target regions in and around the *N4R* gene, and results showed a detection limit of 2 × 10^0^ DNA copies, which is comparable to the gold-standard qPCR method currently used to detect mpox. Particularly, the assay provides results visible to the naked eye within 30 min. This test specifically detects mpox DNA with no cross-reactivity to related DNA viruses including Varicella Zoster Virus (VZV), Hepatitis B virus (HBV), Vaccinia virus (VACV), Herpes simplex virus-1 (HSV-1), Herpes simplex virus-2 (HSV-2), Human papillomavirus-16 (HPV-16) and Human papillomavirus-18 (HPV-18). Furthermore, the LAMP assay has been evaluated using clinical samples from laboratory-confirmed mpox patients and found to be consistent with the qPCR results. Our results show that this single-tube LAMP method can contribute to diagnosis of suspected mpox infections in the field and clinic, especially in regions with limited laboratory resources.

## 1. Introduction

Monkeypox virus (mpox) is a DNA virus of zoonotic origin that was first reported in humans in the Democratic Republic of Congo during 1970 [1,2]. Mpox belongs to the Orthopoxvirus (OPXV) genus of the *Poxviridae* family. Its genome consists of double-stranded DNA approximately 200 kb in length, coding for around 190 genes [3]. Two clades of mpox are known to circulate in different parts of Africa and have divergence in ~0.5% of their genomic sequences [4,5,6]. Infection of humans by mpox is commonly due to close contact with an mpox-infected animal. Transmission between humans can occur through contact with a lesion on the skin or inhalation of large respiratory droplets [7]. The incubation period is 7 to 21 days with typical symptoms including fever, chills and malaise, followed by a centrifugal rash appearing on the palms and soles of the feet [8]. The case fatality rates in humans are estimated to be ~10% or ~3% for the Congo Basin (Central African) clade (Clade I) and the West African clade (Clade IIa), respectively [9]. The actual public health burden of monkeypox is likely underestimated, although outbreaks in Africa have been reported sporadically, usually arising from contact with wildlife reservoirs, particularly in rodents. During the last decades, increased numbers of monkeypox cases have been identified in endemic areas of Central and West Africa: over 19,000 cases between 2000 and 2019 and 15,600 cases between 2021 and 2022 [10,11].

On 7 May 2022, the UK Health Security Agency reported an imported monkeypox case from Nigeria, an endemic country for mpox [12]. As of 7 September 2022, a total of 52,996 laboratory confirmed cases, including 18 deaths, have been reported to the World Health Organization (WHO) [13]. As opposed to past monkeypox outbreaks, which were limited geographically with low case numbers, this is the first time monkeypox cases were reported simultaneously from all six continuously inhabited continents, and in non-endemic regions for mpox [14,15]. On 23 July 2022, the WHO declared the ongoing epidemic of monkeypox to be a Public Health Emergency of International Concern (PHEIC) [16].

Vaccines previously developed for smallpox have been put in use to control monkeypox infections, but supplies are currently limited and the vaccines are estimated to be ~85% effective against mpox [17,18]. Specific vaccines have not yet been developed and approved against mpox. Moreover, tecovirimat (ST-246) and brincidofovir have been approved in the U.S. for treating smallpox. While tecovirimat is more effective than brincidofovir for treating mpox infected cases [19], tecovirimat or brincidofovir can cause drug-resistant mutations under cell culture conditions [20,21]. These results indicate that other drugs should be developed in the future. Rapid and sensitive detection of mpox is a crucial countermeasure to accurately diagnose cases and enact necessary public health policies, such as containment, quarantine and treatment.

The main method for laboratory diagnosis of mpox is the quantitative polymerase chain reaction (qPCR) assay [22,23,24,25]. Due to its high sensitivity, specificity, speed and possibility for high-throughput screening, it is recommended by the WHO as a gold-standard diagnostic method [26]. However, qPCR requires specialized laboratory instruments and skilled staff. In addition, transportation of the collected clinical specimens to the laboratory poses a biosafety risk and can delay results from testing due to logistical hurdles, which has occurred previously with outbreaks for other pathogens, such as the Ebola virus [27]. Therefore, in areas with poor infrastructure and limited medical resources, qPCR assays are not the most suitable for point-of-care testing. More economical and convenient detection methods are needed to accurately detect potential cases of monkeypox on-site in these regions.

Loop-mediated isothermal amplification (LAMP) is a method developed for specific amplification of nucleic acids [28]. This well-established method has several advantages, including high sensitivity, ease of use, speed and low cost. In addition, as the method is performed isothermally at 60–65 °C, a thermocycler (required for qPCR) is not needed for LAMP [29]. There have been numerous applications of LAMP assays for the diagnosis of several pathogens, such as African swine fever virus (ASFV) and SARS-CoV-2 [30,31]. The LAMP reaction can be followed in real-time by visualizing fluorescence through the use of intercalating dyes such as SYTO9 [32]. The reaction also can be visualized by the naked eye via simple color changes based on the production of protons and a subsequent drop in pH resulting from DNA polymerase activity, thus a fluorescence reader (also required for qPCR) is not needed for LAMP [33].

Here, we developed a simple, rapid and sensitive technique for the detection of mpox. In this study, a fluorescent LAMP assay as well as a visual LAMP assay based on the mpox *N4R* gene was successfully developed and validated with clinical samples for mpox detection. Results can be visualized within 1 hour after sample extraction with a sensitivity of 2 × 10^0^ DNA copies, which is comparable to the sensitivity via testing with the qPCR method [24].

## 2. Materials and Methods

### 2.1. LAMP Primer Design and Viral DNA Standard Synthesis

**Figure 1:**
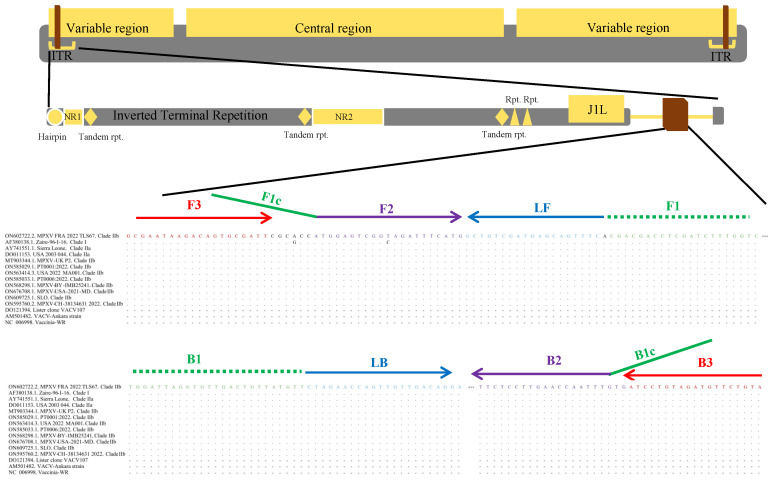
LAMP primer design. Schematic showing location of primer recognition sites around the N4R gene, which is duplicated in left and right inverted terminal repetitions (ITR) of the mpox genome. The positions from 5840nt/190765nt to 6339nt/191264nt are based on the complete genome of MPVX-FRA-2022-TLS67 (GenBank accession: ON602722.2). Outer primers (F3, B3) are indicated by red line arrows, internal primers (BIP and FIP) are indicated by green and purple line arrows, loop primers (LB, FB) are indicated by blue line arrows.

The LAMP primers were designed for detecting the past and present mpox isolates based on phylogenetic analysis of sequences associated with the 2022 epidemic [5,9]. Twelve sequences of mpox including Clade I, Clade IIa, Clade IIb and 3 vaccinia virus strains deposited in NCBI were aligned using MEGA (version 11.0.11) (Figure 1). Two inverted terminal repetitions (positions 5840nt-6339nt/190765nt-191264nt) were identified in the region surrounding the *N4R* gene in the reference mpox strain of this study (MPVX-FRA-2022-TLS67, GenBank accession: ON602722.2) for LAMP primer design. This target region was synthesized and then inserted into the pUC57 plasmid (Sangon Biotech, Shanghai, China) as a template. The primer designs were made utilizing the NEB LAMP Primer Design Tool (version 1.3.0), synthesized commercially by Sangon Biotech (Shanghai, China), and shown in Table S1.

### 2.2. Quantitative PCR

Quantitative PCR assays were conducted on the Light Cycler 96 (Roche, Basel, Switzerland). A set of qPCR primer pairs and probe that target the *G2R* gene was synthesized by Sangon Biotech (Shanghai, China) and the reaction system was prepared as previously described [22]. Briefly, each 20 μL qPCR reaction contained final concentrations of 1x AceQ qPCR Probe Master Mix (Vazyme, Nanjing, China), 0.2 μM for each primer and 200 nM TaqMan probe. The qPCR reactions were performed at 95 °C for 5 min, followed by 40 cycles at 95 °C for 3 s and 60 °C for 30 s. A cycle threshold (Ct) cut-off value less than 34 was defined as positive, based on the Centers for Disease Control and Prevention (CDC) guidelines [34].

### 2.3. LAMP Reaction

The fluorescent LAMP reactions were performed in a 20 μL reaction mixture containing (final concentrations and product ID in brackets): WarmStart Lamp 2x Master Mix (1x, NEB M1700), a mixture of FIP and BIP inner primers (0.8 μM each), a mixture of F3 and B3 outer primers (0.1 μM each), a mixture of LF and LB loop primers (0.2 μM each), SYTO9 (500 nM, Thermo Fisher S34854) and 2 μL of DNA template. The reaction mixture was performed at 65 °C for 60 min in the LightCycler 96 (Roche, Basel, Switzerland) and collected fluorescence signals every minute through the FAM channel. The fluorescent LAMP reactions were considered positive (i.e., Time-to-positive, or Tp) by monitoring the fluorescence signals over a threshold readout when incubated in a qPCR machine.

The visual LAMP reactions were performed in a 20 μL reaction mixture containing (final concentrations and product ID in brackets): WarmStart Colorimetric Lamp 2x Master Mix (1x, NEB M1800), a mixture of FIP and BIP inner primers (0.8 μM each), a mixture of F3 and B3 outer primers (0.1 μM each), a mixture of LF and LB loop primers (0.2 μM each) and 2 μL of DNA template. The reaction was incubated at 65 °C for 60 min in a TGrade Dry Bath Incubator (TIANGEN Biotech, Beijing, China). Positive results were directly visible by the naked eye based on color changes. Due to the decreased pH values caused by positive LAMP reactions, the phenol red pH indicator changed colors from pink to yellow. Positive reactions were indicated by yellow, while negative reactions were indicated by pink. In addition, to evaluate the efficiency of the visual LAMP assay, the LAMP reaction was optimized by testing at different reaction timepoints (10 min, 20 min, 30 min and 40 min) using known concentrations of synthesized viral DNA plasmid standard.

### 2.4. Sensitivity and Specificity of LAMP for the Detection of Mpox

Ten-fold serial dilutions of the DNA plasmid standard ranging from 2 × 10^5^ copies/μL to 2 × 10^0^ copy/μL were used to evaluate the sensitivity of the fluorescent LAMP and the visual LAMP assay for mpox, and each dilution was repeated in triplicate. Concomitantly, the same standards were subjected to qPCR detection following the protocol described above. In addition, the limit of detection (LOD) of the fluorescent and the visual LAMP assay for mpox was analyzed using decreasing concentrations of DNA standard over eight replicates.

The specificity of the fluorescent and the visual LAMP assays was evaluated by using extracted DNA from 7 other DNA viruses including Varicella Zoster Virus (VZV), Hepatitis B virus (HBV), Vaccinia virus (VACV), Herpes simplex virus-1 (HSV-1), Herpes simplex virus-2 (HSV-2), Human papillomavirus-16 (HPV-16) and Human papillomavirus-18 (HPV-18). Viral DNA was extracted by applying the QIAamp DNA Mini Kit (Qiagen, Hilden, Germany) following manufacturer instructions.

### 2.5. Clinical Validation of the LAMP Assay for Monkeypox Virus Detection

Fifteen DNA extractions from biological samples (5 crusts, 9 pus, 1 serum), collected from patients of previous mpox outbreaks in the Central African Republic (CAR), were kindly provided by the Institut Pasteur of Bangui. The detailed information of the samples is shown in Table S2. Five serum samples collected from healthy individuals were used as a negative control. DNA extraction from these clinical samples was performed using the QIAamp DNA Mini Kit (Qiagen, Hilden, Germany) following manufacturer instructions. The DNA samples were stored at −80 °C until use.

To evaluate the performance of the fluorescent and the visual LAMP assays, an equal amount of extracted DNA was added to each 20 μL reaction of the fluorescent LAMP, the visual LAMP and the qPCR assays. The fluorescent LAMP reactions were monitored in real time by Applied Biosystems QuantStudio 1 (Applied Biosystems, Foster City, CA, USA). The qPCR assays were performed following manufacturer instructions using the LightCycler 96 (Roche, Basel, Switzerland).

### 2.6. Generation of Figures and Graphs

All figures and graphs were generated in Adobe Illustrator, v24.1.2 (Adobe, San Jose, CA, USA). The bar graphs and scatter plot were generated using GraphPad Prism 8 (GraphPad Software, San Diego, CA, USA).

## 3. Results

### 3.1. LAMP Primer Design

Based on phylogenetic analysis of mpox sequences, mpox is now divided into three Clades: Clade I (Congo Basin clade), Clade IIa (West African clade) and Clade IIb including the most recent isolates from the 2022 outbreaks [35]. One set of the LAMP primers was found in the highly conserved region (specifically from positions 5840nt/190765nt to 6339nt/191264nt) which are duplicated in left and right inverted terminal repetitions within the viral genome (Figure 1, Table S1).

### 3.2. Sensitivity Evaluation of the LAMP Assay

**Figure 2:**
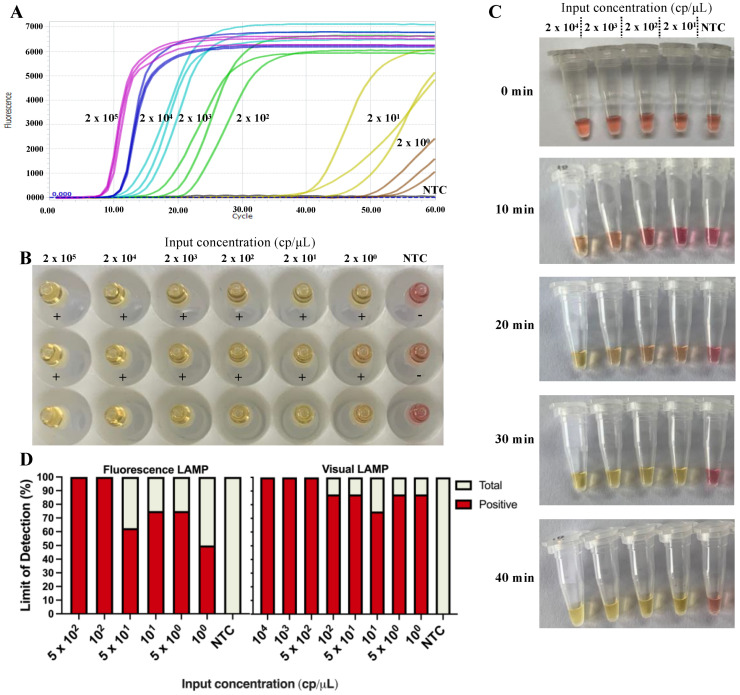
Sensitivity of the mpox LAMP assay as determined using a serially diluted DNA standard plasmid ranging from 2 × 10^5^ to 2 × 10^0^ copies. (**A**) the sensitivity of the fluorescent LAMP assay was monitored by real-time instrument. (**B**) The sensitivity of the visual LAMP assay was evaluated by a change of color from pink to yellow. (**C**) Determination of reaction time of visual LAMP for positive amplification that was assessed using the serially diluted DNA standard plasmid from 2 × 10^4^ to 2 × 10^1^ copies. Observation of color change from pink to yellow indicates a positive reaction. (**D**) Limit of detection (LOD) of the fluorescent LAMP assay was assessed using DNA standard plasmid from 5 × 10^2^ to 1 × 10^0^ copies dilutions in eight repetitions, carried out at 65 °C for 60 min incubation, whereas the LOD of visual LAMP using DNA standard plasmid from 1 × 10^4^ to 1 × 10^0^ copies was assessed in eight replicates, carried out at 65 °C for 60 min incubation. NTC: non-template control.

Ten-fold serial dilutions of mpox DNA standard plasmids, from 2 × 10^5^ to 2 × 10^0^ copies/μL, were used to determine the sensitivity of the fluorescent LAMP assay. As shown in Figure 2A, DNA inputs of 2 × 10^5^ to 2 × 10^0^ copies per 20 μL reaction generated typical amplification curves, whereas the no-template control did not yield an obvious amplification curve, indicating that the fluorescent LAMP can detect as low as 2 × 10^0^ copies per reaction. Importantly, all amplification curves appeared within 20 min and reached a plateau within 30 min when the template input was over 2 × 10^2^ copies. The results were also compared with the qPCR assay (Figure S1A). The standard curve of the qPCR assay for mpox was generated by amplifying the same dilution series of the standard *plasmid*. A good linear relationship between the log of the plasmid copy number and the Ct values was obtained (R^2^ = 0.99) (Figure S1B). In comparison, the sensitivity of fluorescent LAMP (2 × 10^0^ copies per reaction) was observed to be ten-fold higher than that of the qPCR (2 × 10^1^ copies per reaction).

A visual LAMP assay was also developed as a point-of-care test for mpox. The sensitivity of the visual LAMP assay was evaluated by ten-fold serial dilutions of mpox DNA standard plasmids from 2 × 10^5^ to 2 × 10^0^ copies per 20 μL reaction. As shown in Figure 2B, there was no difference in the sensitivity of the visual assay compared to the fluorescent method, indicating that the visual LAMP can also detect as low as 2 × 10^0^ copies per reaction. A successful visual LAMP reaction using phenol red as a pH indicator resulted in color changes from pink to yellow due to the formation of pyrophosphate ions during the period of amplification. Therefore, the DNA standard plasmid, from 2 × 10^4^ to 2 × 10^1^ copies per 20 μL reaction, was used to discern the optimal reaction time for the visual LAMP assay. As shown in Figure 2C, a clear color change of 2 × 10^1^, 2 × 10^2^ and 2 × 10^3^ copies was evident at the 30 min timepoint. Thus, 30 min was selected as the optimal incubation time for the visual LAMP assay.

We then carried out further assessment of the LOD for the fluorescent LAMP and the visual LAMP. As shown in Figure 2D, when the template input was over 100 copies of the DNA plasmid standard, all eight reactions (100%) of the fluorescent LAMP displayed a positive detection, whereas seven reactions (87.5%) of the visual LAMP displayed a positive detection. For 50, 10, 5 copies and 1 copy per reaction, the positive detection rates were 62.5% (5/8), 75% (6/8), 75% (6/8) and 50% (4/8), respectively, for the fluorescent LAMP assay, and 87.5% (7/8), 75% (6/8), 87.5% (7/8) and 87.5% (7/8), respectively, for the visual LAMP assay (Figure S2).

### 3.3. Specificity of the LAMP Assay against Other Large DNA Viruses

**Figure 3:**
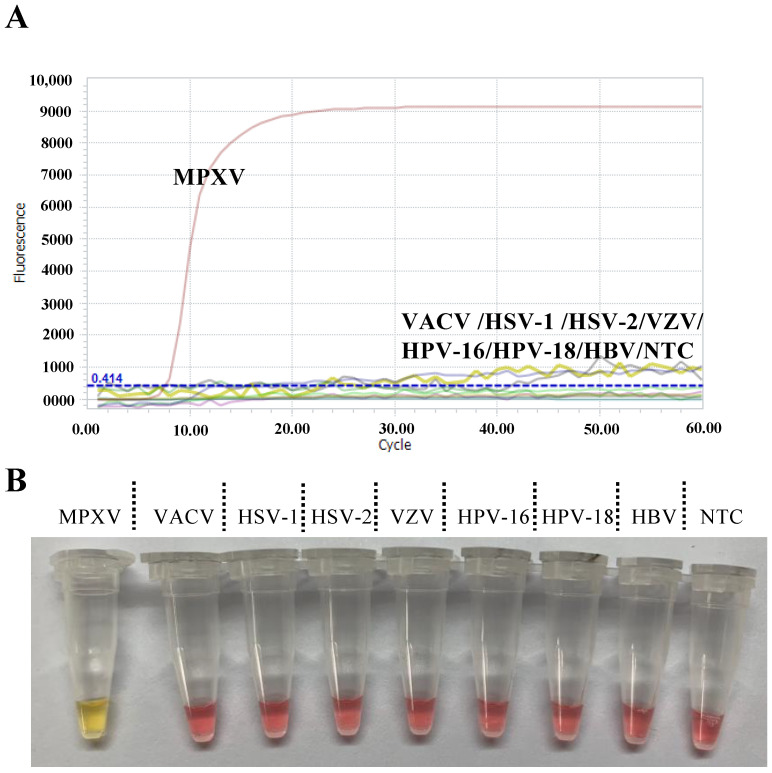
Specificity of the fluorescent LAMP (**A**) and visual LAMP assay (**B**). The DNA viruses used in the assay include: Varicella Zoster Virus (VZV); Hepatitis B virus (HBV); Vaccinia virus (VACV); Herpes simplex virus-1 (HSV-1); Herpes simplex virus-2 (HSV-2); Human papillomavirus-16 (HPV-16); and Human papillomavirus-18 (HPV-18). NTC: non-template control.

We then examined the specificity of the LAMP assays against other common large DNA viruses including Varicella Zoster Virus (VZV), Hepatitis B virus (HBV), Vaccinia virus (VACV), Herpes simplex virus-1 (HSV-1), Herpes simplex virus-2 (HSV-2), Human papillomavirus-16 (HPV-16) and Human papillomavirus-18 (HPV-18). As shown in Figure 3A, there were no amplification curves for all seven large DNA viruses after 60 min of reaction, indicating the high specificity of the fluorescent LAMP. The visual LAMP assay showed the same results which is evidenced by a lack of observed color change after incubation for 60 min (Figure 3B).

### 3.4. Validation of the LAMP Assays Using Clinical Mpox Specimens

**Figure 4:**
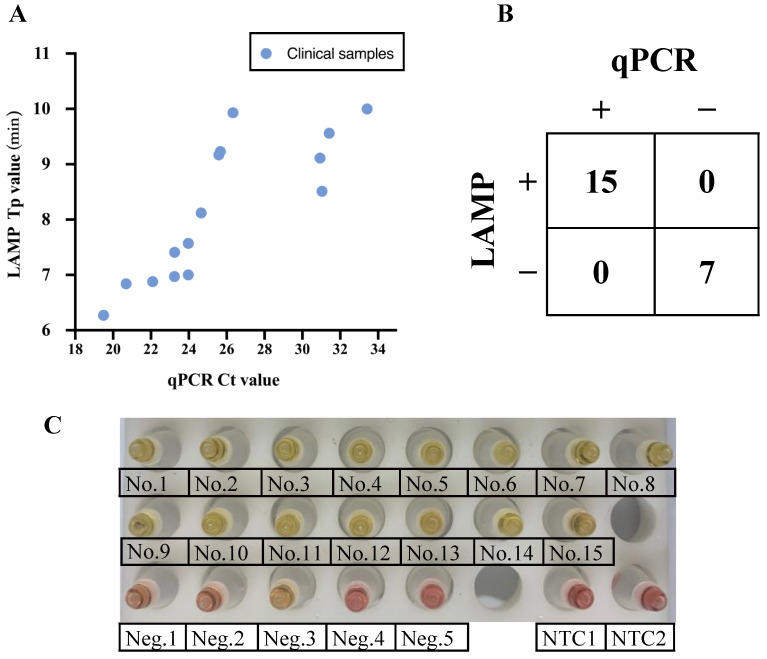
Clinical validation of the mpox LAMP using extracted DNA from fifteen biological samples (five crusts, nine pus, one serum), five healthy serum samples and two non-template controls (NTC). (**A**) Scatter plot of the Tp (time to positive) values of the fluorescent LAMP and the Ct values of the real-time qPCR assay on biological samples. (**B**) Concordance between the fluorescent LAMP assay and qPCR for clinical samples. (**C**) Clinical validation of the visual LAMP was visualized after incubation for 60 min by naked eye.

In order to evaluate the clinical application of mpox diagnosis with these LAMP assays, 15 clinical samples (five crusts, nine pus, one serum) were used from patients of previous mpox outbreaks in the Central African Republic (Table S2). Based on CDC guidelines, a cycle threshold (Ct) cut-off value less than 34 was defined as positive when detecting via qPCR assay. Five serum samples collected from healthy individuals were used as a negative control. An equal amount of clinical sample was added to each 20 μL reaction of the fluorescent and the visual LAMP assays. As shown in Figure 4A, the fluorescent assay showed that all 15 clinical samples were positive and generated signals (Tp value) within 10 min, even for samples with relatively high Ct values. There was 100% (15 in 15) agreement between qPCR (the Ct value of clinical samples ranged from 19.50 to 33.42) and the fluorescent LAMP assay performed on mpox infected samples within 10 min (Figure 4B, Table S2). Similar to fluorescent LAMP, the visual LAMP assays showed that all 15 clinical samples were positive and generated color changes within 30 min (Figure 4C).

## 4. Discussion

More frequently recurring monkeypox outbreaks over the past years have made it necessary to develop a rapid, sensitive and specific diagnostic method for the surveillance of suspected cases. A main reason is because it is challenging to identify monkeypox only based on the clinical symptoms, especially for cases without any clinical presentations. In the absence of smallpox, the main clinical diagnostic obstacle is the discrimination of monkeypox from chickenpox [36]. Moreover, according to the WHO guidelines, any suspected monkeypox cases should be tested in properly equipped laboratories by relevant technical staff under safety regulations. At present, qPCR is the gold standard method for the confirmation of monkeypox virus infection. The challenges to develop robust mpox assays are to find specific, conserved targets due to other OPXV sharing substantial sequence homology to mpox (>90%), which limits the design of the molecular diagnostic assay. In a previous study, Li et al. developed a qPCR assay by using minor groove binding protein-based (MGB) probes to enhance assay sensitivity and specificity [22]. Moreover, both clades (West African and Congo Basin) of mpox have 99% sequence similarity [37], so it is challenging to develop a clade-specific qPCR detection approach due to the limited availability of unique sequences. In an effort to differentiate between isolates from two different clades, the terminal genomic sequences of mpox strains were analyzed [24]. Although sensitive and specific, the qPCR-based mpox detection approach is a time-consuming process and needs specialized instruments and trained staff, which limits their use in the field and clinic without sufficient laboratory support. These limitations need to be overcome. Currently, several isothermal methods have been developed for mpox molecular detection. Davi et al. established a mpox-recombinase polymerase amplification (RPA) assay targeting the *G2R* gene, which produced diagnostic results within 10 min and the results are comparable with qPCR results [38]. Mao et al. developed RPA combined with CRISPR-Cas12a (RPA-Cas12a), real-time RPA, and recombinase-aided amplification (RAA) combined with lateral flow strips (RAA-LFS) against mpox [39]. These assays work by detecting the tumor necrosis factor (TNF) binding protein gene, *G2L* gene and *G2R* gene of the mpox genome, respectively [38,39]. However, RPA can be more expensive than LAMP, because it requires commercial multi-protein reaction mixes as well as chemically modified probes.

Loop-mediated isothermal amplification (LAMP) is another promising isothermal molecular diagnostic technique for virus detection [40]. The LAMP technology relies on a set of six primers: two outer primers (F3 and B3), two inner primers (FIP and BIP), and two loop primers (LF and LB). The reaction is carried out at an isothermal temperature between 60–65 °C. In our study, the target sequences were selected for LAMP assays detecting the terminal repetitions of the genome, which show greater variations among sequences of OPXV strains. After alignment, we selected the LAMP primer sets to identify the conserved region surrounding the *N4R* gene in the MPVX-FRA-2022-TLS67 reference strain. Based on this, we developed the fluorescent LAMP and the visual LAMP detection methods for mpox, which generates accurate results within 30 min. The results of both assays showed 100% consistency with real-time PCR results for mpox detection. The other LAMP assay was used to detect genome of Congo Basin (C-LAMP), West African (W-LAMP) and both Congo Basin and West African (COM-LAMP). The three different LAMP primer sets were designed according to the nucleotide sequences of the Congo Basin-specific *D14L* gene, the West African-specific partial *ATI* gene and the partial *ATI* gene that is shared by both groups. The sensitivity and specificity of COM-LAMP, C-LAMP, and W-LAMP were 80% (45/56) and 100% (64/64); 79% (19/24) and 100% (24/24); and 72% (23/32) and 100% (40/40), respectively [41]. In addition, the recent LAMP assay for mpox detection used the *A27L* and *F3L* genes for designing of the two primer sets. However, the detection limits of these assays were both 20 copies/reaction mixture, which were 10-fold higher in terms of sensitivity, compared with our LAMP assay for mpox detection [42]. It is important to note that the 2022 mpox outbreak has been rapidly spreading in non-endemic countries, mainly in European countries and American countries, which have the resources to provide qPCR testing. However, to prevent future importation of cases, it is important to also enhance and simplify testing in endemic countries of Africa, which do not always have the required resources for laboratory diagnosis. There is an urgent need for modernization of the existing infrastructure and diagnostic facilities. LAMP provides a useful alternative for active surveillance of mpox, since it only requires a heat block and can be directly visualized with the naked eye. In addition, the recommended type of specimen for laboratory detecting monkeypox is skin material and/or swabs of surface lesions. Interestingly, mpox DNA can be found in the saliva of infected cases by using qPCR [43]. Previous reports showed that unpurified SARS-CoV-2 in saliva samples can be detected with no RNA extraction step via a RT-LAMP assay [44,45]. As the reaction temperature of our LAMP assay is 65 °C and mpox virus can be inactivated at 60 °C in under 15 min [46], this LAMP assay can theoretically be used to detect mpox from crude clinical saliva samples without prior DNA extraction. Another limitation in this research is that we only used clinical DNA samples belonging to mpox Clade I to test our LAMP assay, but not those from Clade IIa and Clade IIb. As such, our results could be supplemented with validation of these assays with samples from the 2022 mpox outbreak.

With high sensitivity and specificity, our LAMP assay is simple to use, low cost and time efficient. Moreover, the isothermal LAMP visual method does not require specialized instrument to observe the results. Since the 2022 mpox outbreak has now been classified as a PHEIC by the WHO, our results would extremely strengthen the public health interventions in providing much needed diagnostic services, especially in areas with limited laboratory capacities.

## 5. Conclusions

The LAMP assays developed here enable the rapid and convenient on-site testing of monkeypox in medically under-resourced regions. While validated with Congo Basin clinical samples, the design of the primer theoretically allows for the detection of West African isolates as well. The sensitivity of the assays was shown to be superior to existing qPCR assays, and did not cross-react with sequences from other large DNA viruses or pathogens causing rash/lesions, which is an advantage over previous LAMP assays developed against mpox. While qPCR is still likely to remain the assay of choice due to the ability for quantitation of viral loads and the fact that the 2022 epidemic is occurring primarily in developed countries with the capacity to perform more sophisticated diagnostics, our LAMP assays can play an important role in supplementing, and in some cases substituting, qPCR results in a home or clinical setting.

## Data Availability

Not applicable.

## Supplementary Materials

The following supporting information can be downloaded at: https://www.mdpi.com/article/10.3390/v15010084/s1, Figure S1: Sensitivity of the qPCR assay for mpox detection using DNA standard plasmid; Figure S2: Limit of detection of the mpox visual LAMP assay; Table S1: Primers sequences used for the detection of the mpox via LAMP assay and qPCR; Table S2: Detailed information of mpox biological samples used in this study.
