# Supplementary material for: Development of a Novel Loop-Mediated Isothermal Amplification Method for the Rapid Detection of Monkeypox Virus Infections
Source: Viruses. 2022 Dec 28;15(1):84. doi: 10.3390/v15010084 (PMC9864920; doi:10.3390/v15010084)
Supplement: Supplementary file 1 [file viruses-15-00084-s001.zip › viruses-2103193-supplementary.pdf]

Supplementary Information

Development of a Novel Loop-Mediated Isothermal Amplification Method for the Rapid Detection of Monkeypox Virus Infections

Authors:

Chao Yu[1+], Lulu Zuo[1,2+], Jing Miao[2,3+], Lingjing Mao[2,3], Benjamin Selekon[4], Ella Gonofio[4], Emmanuel Nakoune[4], Nicolas Berthet[3,5*], Gary Wong[1*]

Affiliations:

[1] Viral Hemorrhagic Fevers Research Unit, CAS Key Laboratory of Molecular Virology and Immunology, Institut Pasteur of Shanghai, Chinese Academy of Sciences, Shanghai 200031, China

[2] University of Chinese Academy of Sciences, Beijing 100049, China

[3] Centre for Microbes, Development, and Health, Institut Pasteur of Shanghai, Chinese Academy of Sciences, Unit of Discovery and Molecular Characterization of Pathogens, Shanghai 200031, China

[4] Institut Pasteur of Bangui, Bangui, Central African Republic

[5] Institut Pasteur, Unité Environnement et Risque Infectieux, Cellule d'Intervention Biologique d'Urgence, Paris 75724, France

[+] **Co-first authors**

***Corresponding authors:**

Gary Wong, PhD

Viral Hemorrhagic Fevers Research Unit, CAS Key Laboratory of Molecular Virology and Immunology

Institut Pasteur of Shanghai

Chinese Academy of Sciences

Shanghai, China

E-mail: garyckwong@ips.ac.cn

Nicolas Berthet, PharmD, PhD

Unit of Discovery and Molecular Characterization of Pathogens

The Center for Microbes, Development and Health, CAS Key Laboratory of Molecular Virology and Immunology

Institut Pasteur of Shanghai-Chinese Academy of Sciences,

Shanghai, China

E-mail: nicolas.berthet@pasteur.fr

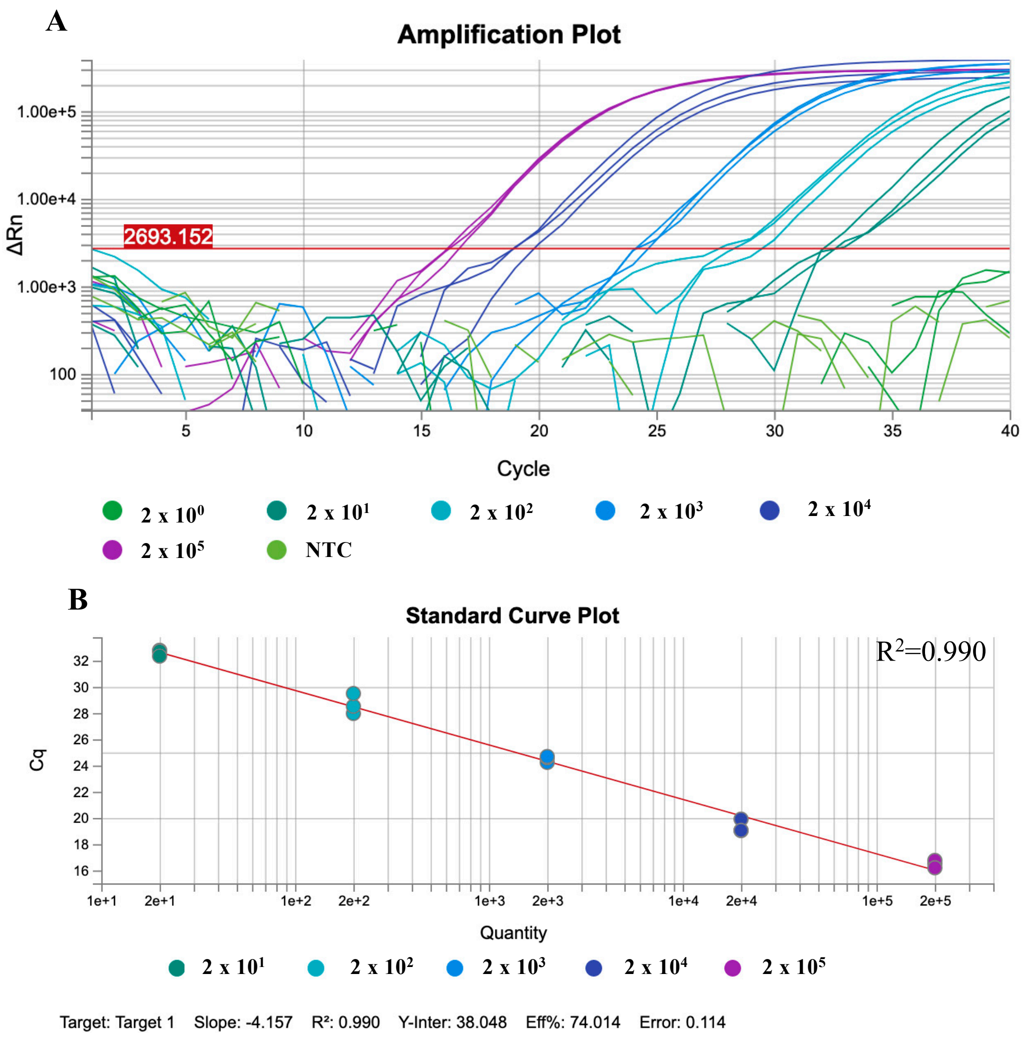

Figure S1. Sensitivity of the qPCR assay for mpox detection using DNA standard plasmid. The plasmid concentrations ranged from 2×10^5 to 2×10^0 copies; non-template control (NTC). (A) amplification curve of the qPCR assay. (B) Standard curve graph generated from serially diluted copies of DNA standard plasmid templates with correlation coefficient value ($R^2 = 0.99$).

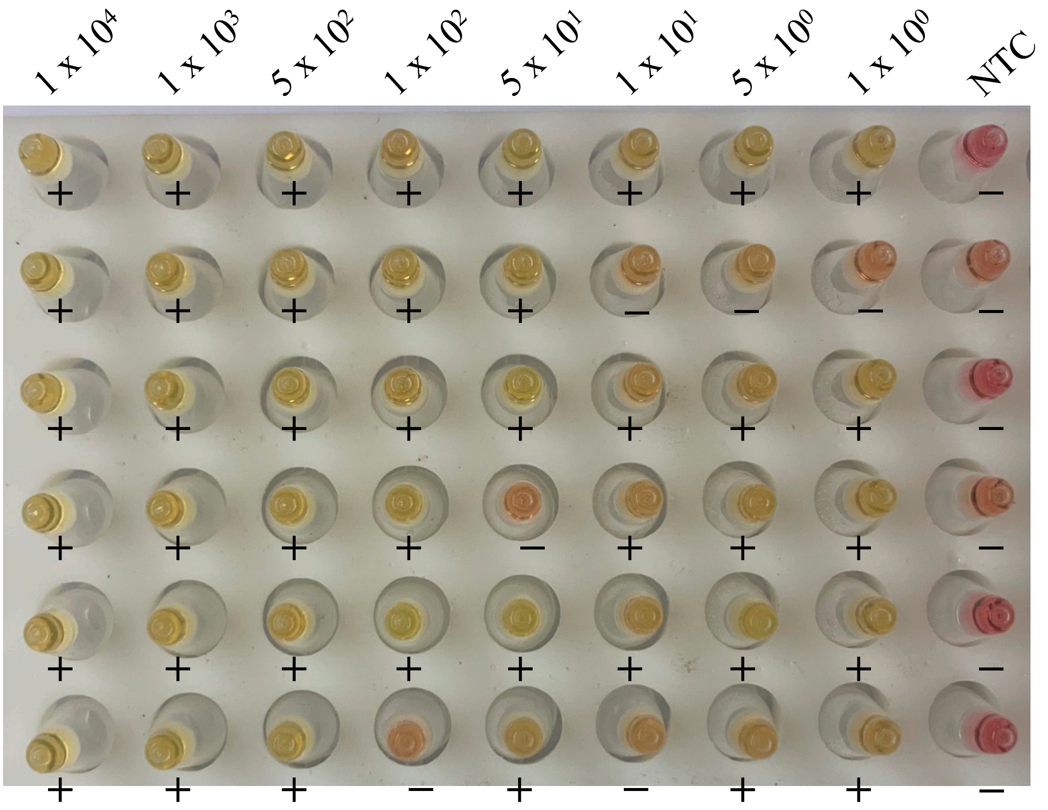

Figure S2. Limit of detection of the mpox visual LAMP assay. The limit of detection was assessed using diluted DNA standard plasmid from 1 × 10^4 to 1× 10^0 copies in 8 replicates. NTC: non-template control.

Table S1. Primer sequences used for the detection of mpox via LAMP assay and qPCR.

Primers	Sequences (5’-3’)	Length (nt)
F3	GCGAATAAGACAGTGCGATT	20
B3	TCATACAGAACATCTACAGGAT	22
FIP	GACCAAAGATCGAGGTCGTCGATGGAGTCGGTAGATTTCATG	42
BIP	TGGATTAGGTGTTGACTGTTATGTTCACAAATTGGTTCAAGGAGAA	46
LF	GAAACTGCTCATCGACAGC	19
LB	CTAGAACCAGTTGTTGACAGGA	22
Forward	GGAAAATGTAAAGACAACGAATACAG	27
Reverse	GCTATCACATAATCTGGAAGCGTA	24
Probe	FAM-AAGCCGTAATCTA<BHQ-1dT>GTTGTCTATCGTGTCC-Spacer C6	30

Table S2. Detailed information of mpox biological samples used in this study.

Sample ID	Sample Type	Real-time qPCR (Ct)		LAMP (Tp)
1	Crusts	20.69	+	6.84
2	Pus	33.42	+	10.00
3	Crusts	26.33	+	9.93
4	Crusts	25.59	+	9.17
5	Crusts	31.04	+	8.51
6	Crusts	23.25	+	7.41
7	Pus	23.63	+	7.00
8	Pus	25.67	+	9.23
9	Pus	30.93	+	9.11
10	Pus	22.09	+	6.88
11	Pus	24.66	+	8.12
12	Pus	19.50	+	6.27
13	Pus	23.24	+	6.97
14	Pus	23.98	+	7.57
15	Serum	31.42	+	9.56
